# Natural Astaxanthin Is a Green Antioxidant Able to Counteract Lipid Peroxidation and Ferroptotic Cell Death

**DOI:** 10.3390/ijms232315137

**Published:** 2022-12-01

**Authors:** Nicola Rizzardi, Laura Pezzolesi, Chiara Samorì, Federica Senese, Chiara Zalambani, Walter Pitacco, Natalia Calonghi, Christian Bergamini, Cecilia Prata, Romana Fato

**Affiliations:** 1 Department of Pharmacy and Biotechnology, Alma Mater Studiorum-University of Bologna, 40126 Bologna, Italy; 2 Department of Biological, Geological, and Environmental Sciences, Alma Mater Studiorum-University of Bologna, Campus Ravenna, 48121 Ravenna, Italy; 3 Department of Chemistry “G. Ciamician”, Alma Mater Studiorum-University of Bologna, Campus Ravenna, 48121 Ravenna, Italy

**Keywords:** astaxanthin, *Haematococcus pluvialis*, oxidative stress, lipoperoxidation, ferroptosis, green chemistry

## Abstract

Astaxanthin is a red orange xanthophyll carotenoid produced mainly by microalgae but which can also be chemically synthesized. As demonstrated by several studies, this lipophilic molecule is endowed with potent antioxidant properties and is able to modulate biological functions. Unlike synthetic astaxanthin, natural astaxanthin (NAst) is considered safe for human nutrition, and its production is considered eco-friendly. The antioxidant activity of astaxanthin depends on its bioavailability, which, in turn, is related to its hydrophobicity. In this study, we analyzed the water-solubility of NAst and assessed its protective effect against oxidative stress by means of different approaches using a neuroblastoma cell model. Moreover, due to its highly lipophilic nature, astaxanthin is particularly protective against lipid peroxidation; therefore, the role of NAst in counteracting ferroptosis was investigated. This recently discovered process of programmed cell death is indeed characterized by iron-dependent lipid peroxidation and seems to be linked to the onset and development of oxidative-stress-related diseases. The promising results of this study, together with the “green sources” from which astaxanthin could derive, suggest a potential role for NAst in the prevention and co-treatment of chronic degenerative diseases by means of a sustainable approach.

## 1. Introduction

**Figure 1:**
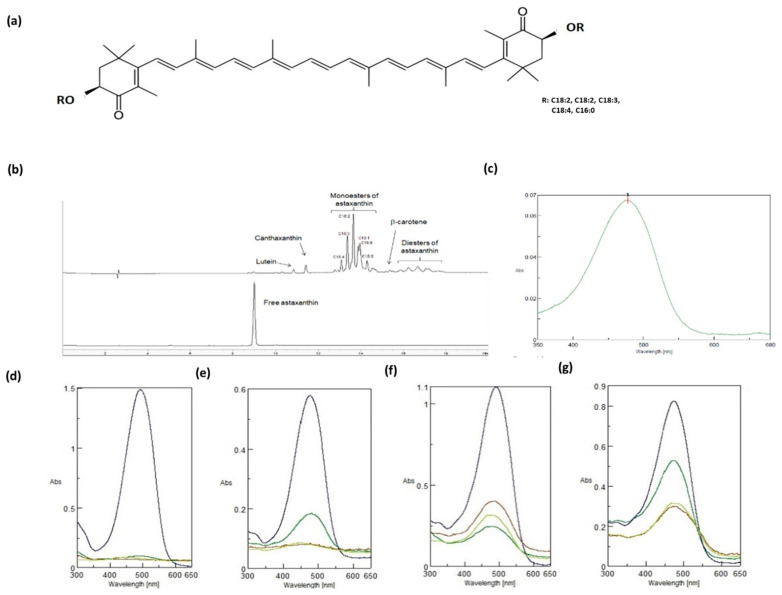
**Physical–chemical characteristics of natural astaxanthin (NAst).** (**a**) NAst structure and its monoesters. (**b**) HPLC chromatogram of carotenoid-enriched extract from *Haematococcus pluvialis* (up) and synthetic astaxanthin (down). (**c**) UV-vis spectrum in ethanol of NAst extracted and purified from *H. pluvialis*. (**d**) UV-vis spectra of synthetic astaxanthin in different DMSO/water mixtures. (**e**) UV-vis spectra of synthetic astaxanthin in different EtOH/water mixtures. (**f**) UV-vis spectra of NAst in different DMSO/water mixtures. (**g**) UV-vis spectra of NAst in different EtOH/water mixtures. Black traces are for 0% water, dark green traces are for 25% water, red traces are for 50% water, and light green traces are for 100% water.

Astaxanthin (3,3′-dihydroxy-β, β′-carotene-4,4′-dione) is a lipophilic xanthophyll carotenoid with a molecular weight of 596.8 Da and the molecular formula C_40_H_52_O_4_. Its structure consists of a chain of conjugated double bonds (a polyene chain) and two polar ionone rings at both ends of the molecule (Figure 1a). This chemical structure confers a red-orange colour, together with general metabolic and physiological activities typical of carotenoids. However, astaxanthin has unique properties, probably due to the presence of the hydroxyl and keto moieties on each ionone ring [1,2], which allow astaxanthin to be inserted into the membrane and to span its entire width. Finally, astaxanthin exists in two predominant forms, unesterified (found in yeast or of synthetic origin) and esterified (found in *algae*), with different fatty acids, the compositions of which depend on the organism of origin and growth conditions.

Numerous studies on astaxanthin have demonstrated its potent antioxidant activities both in vitro and in vivo, such as a quenching effect on singlet oxygen; powerful scavenging effects on superoxide, hydrogen peroxide, and hydroxyl radicals; and an inhibitory effect on lipid peroxidation [3,4,5,6]. As a result, astaxanthin is able to modulate biological functions related to lipid peroxidation, resulting in beneficial effects in terms of prevention and co-treatment of chronic degenerative diseases, such as cardiovascular and neurodegenerative diseases, macular degeneration, and cancer [7,8,9,10,11,12,13,14]. Recent studies suggest that natural astaxanthin (NAst) exhibits higher antioxidant and protective activities than other common carotenoids, including its synthetic form [2,9,15,16,17,18,19,20,21]. Microalgae, its primary bio-synthesizers [20], enhance the reddish-pink colours of the animals that feed on them, such as shrimps, crawfish, crabs, lobsters, trout, and salmonids [22]. It is noteworthy that natural astaxanthin has been approved by the United States Food and Drug Administration (USFDA) as a pigment for use in the aquaculture industry [18,22] and is considered a natural food dye by the European Commission [23,24,25,26].

Although most of the astaxanthin available on the market is produced synthetically, the growing interest in green chemistry and the green economy is leading to the valorization of natural astaxanthin.

Organisms able to produce astaxanthin include the marine *Agrobacterium aurantiacum* [27,28], the red yeast *Phaffia rhodozyma* [6], the microalgae *Chlorococcum sp* and *Chlorella zofingiensis* [29], and, in particular, the green microalga *Haematococcus pluvialis* (*H. pluvialis*), which is able to accumulate large amounts (1.5–5% by dry weight) of the pigment [30,31,32] under specific stressors when a red-cyst phase is induced. *H. pluvialis* is the most accepted organism for the production of NAst [6,16,19,32,33,34,35], an extraction process having been developed on an industrial scale in the late 1990s [35]. Red cysts have a robust cell wall; thus, energy-intensive cell-disruption approaches are required to enhance the recovery of astaxanthin from the cells.

Taking into consideration the numerous beneficial properties attributed to this carotenoid [3,5,6,36], this study aims to elucidate the solubility and in vitro antioxidant activities of NAst, using a well-established neuronal cell model (SHSY-5Y) [37,38,39,40,41]. We choose natural astaxanthin since it has a higher antioxidant capacity, is safer, and has a lower environmental impact than the synthetic form. The antioxidant effects of NAst were evaluated in terms of abilities to decrease intracellular ROS levels, protect against lipid peroxidation, and increase phase II antioxidant enzyme NQO1 activity. The capacity of NAst to increase Nrf2, glutathione-disulfide reductase (GSR), thioredoxin reductase 1 (TXNRD1), and catalase transcription has been investigated [42]. This study investigates for the first time the potential protective role of NAst against ferroptosis—a type of cell death caused by excessive iron-dependent lipid peroxidation closely related to the pathophysiological processes of many diseases [43,44,45,46].

## 2. Results

### 2.1. Physical–Chemical Characteristics of NAst

The carotenoid-enriched extract resulted in 43 ± 2 wt% of biomass and was composed of 5.9 ± 0.2 wt% NAst, corresponding to 2.5 ± 0.1 wt% of NAst in the biomass. The chromatogram of the extract obtained by HPLC UV-Vis at 470 nm was predominantly characterized by peaks of astaxanthin monoesters, while in the synthetic astaxanthin chromatogram only the free form was present. Esters identified by LC-MS on the basis of molecular weight were found to be mainly monoesters of linoleic (C18:2) and linolenic acids (C18:3), followed by oleic (C18:1) and stearidonic acid (C18:4) monoesters, while the palmitic acid (C16:0) monoester was the only C16 ester detected. Minor peaks found in the extract were due to astaxanthin diesters, lutein, canthaxanthin, and b-carotene (Figure 1b). The UV-vis absorption spectrum of NAst presented a peak at 478 nm and a small secondary peak at 670 nm corresponding to the chlorophyll present in the extract [47], as shown in Figure 1c. To test and compare the water-solubility of NAst and synthetic astaxanthin, we measured the UV-vis spectra in different DMSO/water and ethanol/water mixtures and found that NAst was more water-soluble than the synthetic astaxanthin (Figure 1d–g). These latter data suggest that NAst may be more bioavailable towards the synthetic molecule.

### 2.2. Antioxidant Effects of Natural Astaxanthin

Astaxanthin is a potent antioxidant; however, its poor solubility in water hinders its distribution in cells and thus decreases its ability to scavenge reactive oxygen species (ROS). Promising results concerning NAst solubility prompted us to evaluate its antioxidants effects in a neuroblastoma cell model.

**Figure 2:**
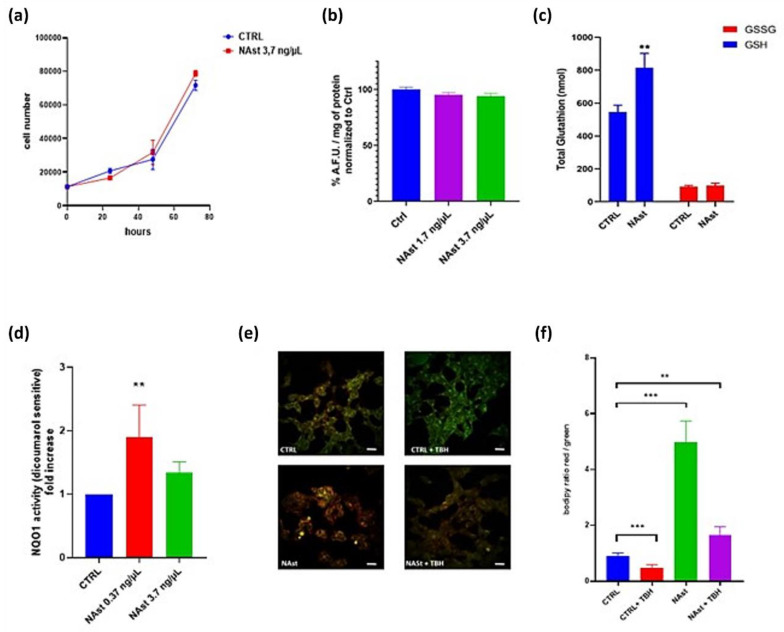
**Antioxidant activities of NAst.** (**a**) Proliferation of SHSY-5Y cells supplemented with 3.7 ng/µL of NAst and synthetic astaxanthin. Cells were counted in triplicate by the Trypan blue exclusion method. Data are reported as cell numbers ± SDs. (**b**) Effects of NAst on intracellular ROS levels in SHSY-5Y cells. Cells were supplemented with different amounts of NAst for 24 h. Intracellular ROS levels were measured in live cells using the probe DCFH-DA by means of spectrofluorimetric analysis. Data are reported as percentages in comparison to controls ± SEMs. (**c**) Total glutathione evaluation in SHSY-5Y cells supplemented with 3.7 ng/μL NAst by luminescent assay. Data are shown as the means ± SDs of at least three independent experiments. ** *p* ≤ 0.01. (**d**) NQO1 activity determination in lysates from SHSY-5Y cells supplemented with NAst for 24 h. Dicoumarol was used to specifically inhibit the enzyme. Data are reported as fold increases in comparison with untreated cells ± SDs; *n* = 4. ** *p* ≤ 0.01. (**e**) Representative micrographs of SHSY-5Y cells stained with the lipid peroxidation sensor BODIPY C11. Cells were supplemented with 3.7 ng/µL of NAst for 24 h. Oxidative stress was induced using 130 µM of TBH for 30 min. Scale bars: 10 µm. (**f**) Quantitative analysis of the red/green BODIPY C11 fluorescence ratio performed using ImageJ software. Five randomly chosen fields were analyzed for each condition. Error bars indicate SDs. ** *p* ≤ 0.01, *** *p* ≤ 0.001.

In order to rule out potential cytotoxic effects, a human neuroblastoma cell line (SHSY-5Y) was incubated with 3.7 ng/µL Nast, and cell proliferation was followed for 72 h without observation of any alteration in cell growth (Figure 2a).

Then, the antioxidant effect of the molecule was evaluated using the fluorescent probe 2′,7′-dichlorodihydrofluorescein diacetate (DCFH-DA). Basal ROS levels in cells treated with NAst for 24 h were not significantly altered with respect to controls (Figure 2b). The absence of intracellular antioxidant activity can be attributed to the lipophilicity of this molecule.

**Figure 3:**
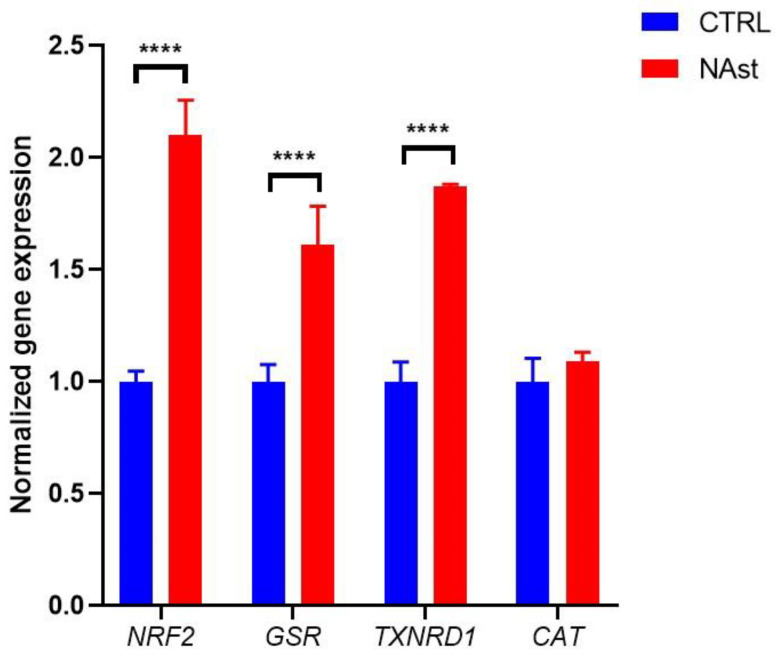
**Normalized gene expression of NFE2L2 (Nrf2), GSR, TXNRD1, and CAT, analyzed by RT-PCR.** Data are expressed as means of the relative expressions (fold changes) between the NAst treatments and the control samples. Data are shown as the means ± SDs of at least three independent experiments. **** *p* ≤ 0.001 vs. control.

Since glutathione is the antioxidant that is most abundant at the intracellular level, its concentration in the presence or absence of NAst was measured. The obtained results showed that the total amount of glutathione significantly increased in the cells incubated with 3.7 ng/µL NAst for 3 h compared with control cells. In particular, the reduced form of glutathione was significantly higher in the presence of NAst (Figure 3c).

The redox homeostasis of cells ensures the balance between oxidizing and reducing reactions, which are catalyzed by a variety of antioxidant enzymes. Therefore, we also evaluated whether cell treatment with NAst modulates the activity of the phase II antioxidant-inducible enzyme NQO1. NQO1 activity was significantly increased in SHSY-5Y cells after treatment with NAst (Figure 2d), suggesting that, in addition to its direct antioxidant effect due to its chemical structure, the treatment with the molecule may increase the cell’s antioxidant defences.

Moreover, taking into consideration the lipophilicity of astaxanthin, the lipid peroxidation sensor BODIPY C11 was used in order to assess whether NAst could protect the membranes from oxidative stress damages. BODIPY C11 is a lipophilic fluorescent probe used to detect reactive oxygen species (ROS) in membranes. Oxidation of the polyunsaturated butadienyl portion of the dye results in a shift of the fluorescence emission peak from red to green. We measured the BODIPY C11 signal in SHSY-5Y cells incubated with NAst after exposure to the pro-oxidant molecule tert-butyl hydroperoxide (TBH). The results showed that cell treatment with NAst significantly decreased lipid peroxidation, both in basal conditions and in TBH-exposed cells (Figure 2e,f).

To further analyze the mechanisms responsible for the antioxidant activities of NAst, the modulation of Nrf2 expression was evaluated as well as that of some downstream genes involved in glutathione metabolism (GSR, TXNRD1) and catalase (CAT)—an antioxidant enzyme regulated by Nrf2 but not directly involved in glutathione metabolism.

The results obtained by the RT-PCR analysis showed that, in cells treated with Nast, a significant increase in the expression of Nrf2, GSR, and TXN occurred in comparison to control cells, while catalase expression was unchanged.

### 2.3. Natural Astaxanthin Protects SHSY-5Y Cells from Ferroptosis

**Figure 4:**
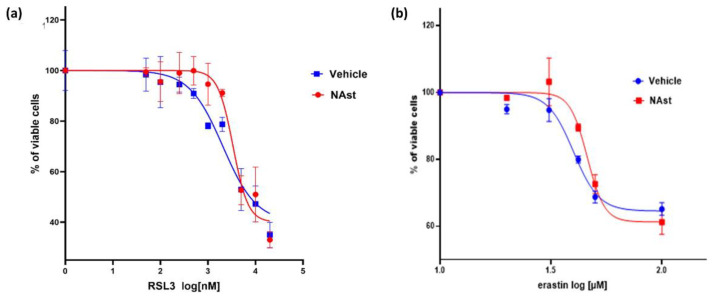
**Viability of SHSY-5Y cells in the presence of different concentrations of the ferroptosis inducers RSL3 and erastin.** Cells were supplemented with 3.7 ng/µL of NAst or vehicle for 24 h and subsequently treated for 24 h with different concentrations of the ferroptosis inducers RSL3 (**a**) and erastin (**b**). Data are reported as the percentages of viable cells ± SDs in comparison to controls; *n* = 3. Cell viability was determined by the MTT assay, as described in the Material and Methods section.

Ferroptosis is a type of iron-dependent cell death characterized by the accumulation of lipid peroxides [44]. Therefore, we evaluated whether incubation with NAst could protect SHSY-5Y cells against this oxidative process. To this purpose, two different ferroptosis inducers were used: RSL3, which inhibits the glutathione peroxidase 4 (GPX4) enzyme—an essential regulator of ferroptosis [45]—and erastin, which induces depletion of reduced glutathione by inhibiting the cell-surface cysteine–glutamate antiporter (system Xc-) [46]. Cell proliferation after ferroptosis induction was assessed by MTT assay. Figure 4a shows that the IC_50_ value for RSL3 in the SHSY-5Y control cells was 2.3 µM, whereas after incubation for 24 h with NAst the IC value increased to 3.4 µM. Using erastin, similar behaviour was observed: the IC_50_ value for RSL3 increased from 39 µM in control cells to 46 µM in cells incubated with NAst (Figure 4b).

**Figure 5:**
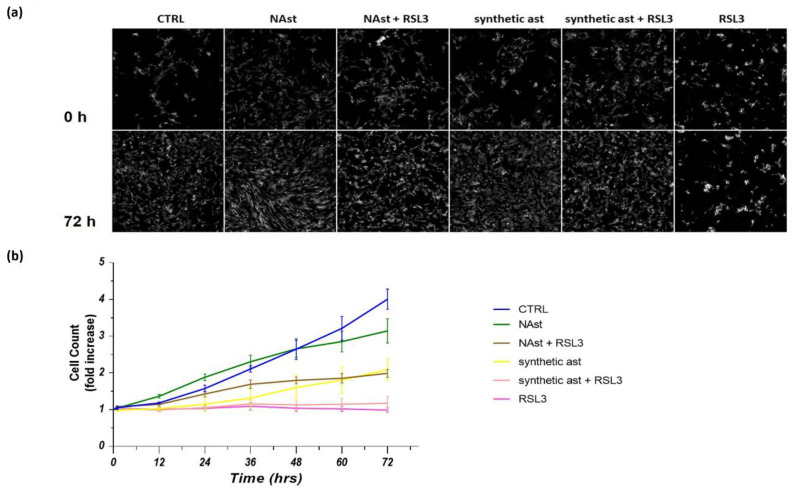
**Quantitative phase imaging (QPI) and SHSY-5Y cell proliferation analysis.** (**a**) Representative images of cells pretreated for 24 h with natural astaxanthin (NAst), synthetic astaxanthin (ast), and vehicle (ctrl), acquired at 0 h and after 72 h of incubation with RSL3. (**b**) Plot of cell count over time. Coloured lines are for control cells (blue line) and for cells treated with synthetic astaxanthin (yellow line), synthetic astaxanthin and RSL3 (pink line), NAst (green line), NAst and RSL3 (brown line), and RSL3 alone (purple line), for up to 72 h. Each time point represents the average of five independent wells ± SEMs. Scale bar: 200 µm.

The biological effects of NAst and synthetic astaxanthin (ast) in SHSY-5Y cells treated with the ferroptotic inducer RSL3 were further investigated using Livecyte technology, which allows simultaneous monitoring of changes in proliferation, motility, and morphology. Figure 5a reports the images of control and treated cells after 0 and 72 h of growth. Figure 5b shows the cell growth plot, highlighting important differences between synthetic ast and NAst. Indeed, synthetic ast decreased cell numbers after just 12 h of treatment, while NAst had no effect on cell growth up to 72 h. The treatment with RSL3 induced a cytotoxic effect leading to cell proliferation arrest. The pretreatment with synthetic ast or NAst protected cells from the cytotoxic insult. Interestingly, NAst seems more effective than synthetic ast in protecting cells from ferroptotic induction.

**Figure 6:**
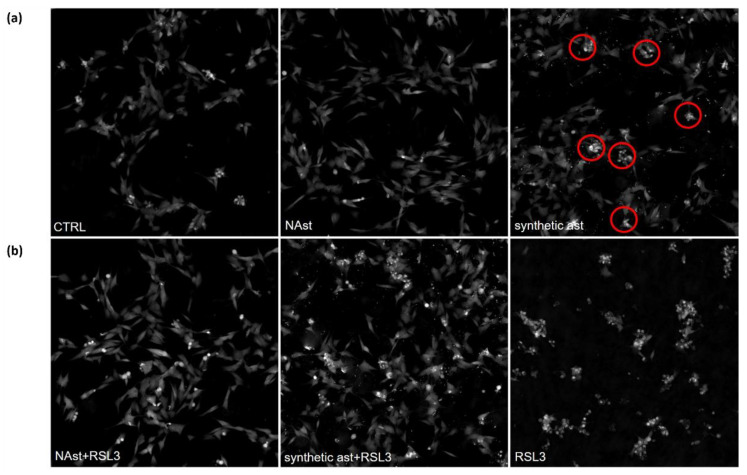
**Quantitative phase images of RSL3-treated SHSY-5Y cells in the presence of synthetic and natural astaxanthin.** (**a**) Representative images of SHSY-5Y cells pretreated for 24 h with NAst, synthetic ast, and vehicle (ctrl) acquired after 12 h of Livecyte monitoring. Red circles indicate dense cells. (**b**) Representative images of SHSY-5Y cells pretreated for 24 h with NAst, synthetic astaxanthin, and vehicle acquired after 12 h of incubation with RSL3. Scale bar: 200 µm.

The different effects of natural and synthetic astaxanthin treatments were visible after just 12 h, as shown in Figure 6a. In fact, treatment with NAst did not modify the morphologies of the cells, which remained elongated. On the other hand, the exposure of SHSY-5Y cells to synthetic Ast caused the loss of the typical elongated morphology characteristic of the control cells and they became smaller and rounder than the controls. In addition, the presence of dense cells (indicated by red circles) suggested a slight induction of cell death.

Treatment with RSL3 induced a strong cytotoxic effect, as shown in Figure 6b. The pretreatment with natural or synthetic astaxanthin showed a protective effect; strikingly, NAst exerted a higher protective effect than the synthetic one, as evidenced by the lower number of small and thickened cells. To deeply investigate this phenomenon, some motility parameters were analyzed, such as instantaneous velocity, track speed, and displacement.

**Figure 7:**
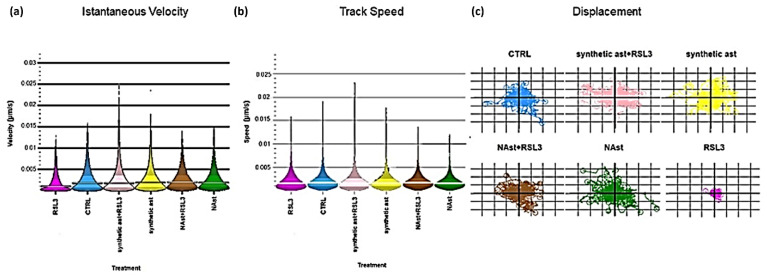
**Analysis of cell motion parameters.** (**a**) Instantaneous velocity, (**b**) track speed, and (**c**) displacement analyses over 72 h of SHSY-5Y cells under different treatment conditions, as reported in the Materials and Methods section. Error bars in (**a**) and (**b**) represent inter-quartile ranges; *n* = 5.

As shown in Figure 7a,b, cells exposed to RSL3 presented lower mobility in comparison to the controls; when cells were pretreated with natural and synthetic astaxanthin, the exposure to RSL3 did not induce any changes in cell motion parameters.

Moreover, plots of cell displacement for random migration studies were obtained in the same conditions. This metric represents the distance a cell migrates relative to its point of origin and also considers the degree to which a cell meanders from its starting point to its end point. The cell displacement graphs shown in Figure 7c suggested that SHSY-5Y cells pretreated with natural or synthetic astaxanthin and exposed to RSL3 did not migrate farther from their points of origin than the control cells. On the contrary, RSL3-treated cells, without astaxanthin preincubation, showed lower displacement, indicating an altered mobility pattern.

## 3. Discussion and Conclusions

In the last decade, studies on astaxanthin as a potent antioxidant and anti-inflammatory agent have shown encouraging results. Natural astaxanthin (NAst) has been proven to be a more powerful antioxidant than other common carotenes [20,21]. In *Haematococcus* p., NAst occurs in three different forms—free (5%), fatty acid monoesters (70%), and fatty acid diesters (25%)—while synthetic astaxanthin occurs 100% in the free form. Furthermore, from a stereochemical point of view, synthetic astaxanthin is all in the *trans* form, while 10% of NAst is in the *cis* form. Differences in chemical structure and stereochemistry might be responsible for the superior antioxidant properties of natural astaxanthin as compared with synthetic astaxanthin. We can speculate that NAst exhibits better antioxidant activity than synthetic astaxanthin when administered to cultured cells because of its better solubility in water. By measuring the astaxanthin UV-Vis spectra for different solvent mixtures (DMSO/H_2_O and EtOH/H_2_O), we showed that NAst is more soluble than the synthetic form, even when the solvent mixture has a high water content.

To test the antioxidant properties of NAst, we initially used a human neuroblastoma cell model (SHSY-5Y) and two different oxidative stress probes: DCFH-DA and BODIPY C11 [48]. The results showed that NAst was effective in reducing the cellular level of peroxides. However, the most pronounced protective effect was observed using the lipid peroxidation sensor, BODIPY C11. NAst treatment protected membrane lipids from peroxidation under both basal conditions and following treatment with the prooxidant molecule TBH. On the other hand, when using DCFH-DA—an ROS sensor that, after deacetylation, becomes fluorescent when oxidized by intracellular peroxides—the protective effect of NAst was less pronounced. This behaviour can be explained by considering the highly lipophilic nature of the molecule, which favours its distribution in lipophilic compartments, such as cell membranes, and therefore makes the molecule particularly protective against lipid peroxidation.

For these reasons, we tested NAst’s ability to counteract a particular type of cell death called ferroptosis, characterized by iron-dependent lipid peroxidation [44]. Ferroptosis is a recently identified mechanism of regulated cell death caused by the redox state disorder in the intracellular microenvironment controlled by GSH and glutathione peroxidase 4 (GPX4). Moreover, ferroptosis is inhibited by iron chelators and lipophilic antioxidants [43,44,49], including vitamin E, Coenzyme Q, ferrostatin-1 (Fer-1), liproxstatin-1 (Lip-1), and possibly potent bioactive polyphenols. Noticeably, ferroptosis is linked to the pathophysiological processes of various diseases, such as tumours, nervous system diseases, ischemia–reperfusion injury, kidney injury, and blood diseases. We induced ferroptosis in our cell model using RSL3 and erastin, which, through two different mechanisms, interfere with the level and utilization of glutathione, resulting in reduction in cell antioxidant capacity and consequently cell death [45,46]. Pre-treatment with NAst was able to significantly increase the IC_50_ values of both ferroptosis inducers in SHSY-5Y cells, suggesting a protective effect. In order to deeply analyze the protective effect of NAst against ferroptosis, we compared its activity with that of synthetic astaxanthin by exploiting quantitative phase image analysis. This innovative approach allowed us to monitor over three days the cell growth, morphology, and mobility parameters in different conditions. The results clearly indicated better anti-ferroptotic activity for NAst compared to synthetic astaxanthin, which could be attributed to the higher water-solubility of NAst, together with a possible synergistic effect exerted by other bioactive compounds present in the natural extract.

The antioxidant properties of astaxanthin are strictly related to its chemical structure. In addition, recent studies have shown that astaxanthin can increase the nuclear localization of Nrf2 and consequently increase the expression of phase II antioxidant enzymes [42]. Therefore, the effects of NAst on different players involved in the cellular antioxidant system was evaluated. We report that NAst was able to increase the intracellular concentration of glutathione—the most abundant endogenous antioxidant molecule—in particular, its reduced form. Moreover, we observed that NAst increased the transcription of Nrf2 and consequently the transcription of glutathione–disulfide reductase (GSR) and thioredoxin reductase 1 (TrxR1). Nrf2 is a transcription factor that plays a pivotal role in the activation of a large number of cell protection genes involved in antioxidant defence and redox signalling in different situations, such as ferroptosis [50]. The GSR gene encodes a member of the class-I pyridine nucleotide-disulfide oxidoreductase family. This enzyme is a homodimeric flavoprotein and is a key enzyme in cellular antioxidant defence, as it reduces oxidized glutathione disulfide (GSSG) to the sulfhydryl form GSH. Thioredoxin reductase 1 (TrxR1) catalyzes the NADPH-dependent reduction of the disulfide in thioredoxin, which mediates a wide range of functions in cellular proliferation, defence against oxidative stress, apoptosis, and redox control. Thioredoxin, in turn, alleviates oxidative stress by scavenging reactive oxygen species (ROS), regulates a variety of redox-sensitive signalling pathways as well as ROS-independent genes, and exerts cytoprotective effects.

As recently reported [50], Nrf2 plays a central role in upregulating anti-ferroptotic defence. Nrf2 can directly or indirectly regulate GPX4 expression and function, as well as the expression of several enzymes involved in GSH synthesis and other oxidoreductases, such as thioredoxin reductase (TXNRD1), that use GSH and NADPH to reduce oxidative substrates [51]. Therefore, NAst could counteract ferroptosis through two independent mechanisms: (1) direct antioxidant activity at plasma-membrane level and (2) via induction of the antioxidant pathway mediated by Nrf2.

In this context, the induction of Nrf2 by NAst accounts for the increased activity of NQO1—a phase II antioxidant enzyme—observed in cells treated with NAst.

Taken together, our data are in good agreement with those obtained by several research groups who have studied the effects of astaxanthin in both in vitro and in vivo experimental models [18,52,53]. As recently reported by Zhang et al., astaxanthin is a potent activator of the PI3K/Akt signalling pathway in SH-SY5Y cells, upregulating Nrf2 and HO-1 [54]. In the same cell model, Wang et al. reported that astaxanthin increased the levels of HO-1, thus acting cytoprotectively against ß-amyloid peptides (Aß25-35) [55]. Astaxanthin is a non-provitamin A carotenoid and is not thought to undergo retinol conversion; however, some studies have suggested that astaxanthin could be involved in RAR or retinoid signalling [56,57,58]. Astaxanthin might act via RAR or other nuclear receptors to modulate the activities of antioxidant enzymes. Nevertheless, the exact mechanism underlying this NAst effect still remains unclear.

In summary, the peculiar chemical–physical characteristics and activities of NAst suggest that its supplementation could represent a potential novel sustainable approach for counteracting and/or co-treating pathologies, including chronic degenerative diseases, characterized by oxidative stress and ferroptosis [59,60]. As far as safety is concerned, it is noteworthy that NAst has been generally recognized as safe (GRAS). It can be sold as a dietary supplement and has been approved as a food colourant (E161j) by the European Commission for use in the food and beverage industries [26,61,62].

Finally, considering the promising properties of NAst, we highlight the importance of improving and enhancing the extraction processes from natural sources from the perspectives of green chemistry, sustainability, and market competitiveness.

## 4. Materials and Methods

### 4.1. Algal Cultivation

*Haematococcus pluvialis* (strain HP5, isolated in Ravenna in 2014) was cultured in 1.5 L flasks using CHU13 medium [63] and grown in the green vegetative phase at a temperature of 21 ± 1 °C, with a light intensity of 100 μmol of photons per m^2^ per s and a 16 h light:8 h light/dark cycle. After 25–30 days, cultures were stressed under a high light intensity (450–500 μmol of photons per m^2^ per s) at 25 ± 2 °C and nutrient starvation at a 3-times dilution of the algal culture. Mature aplanospores (red cysts) were obtained and after 30–40 days were used for the astaxanthin extraction.

### 4.2. Astaxanthin Analysis

The red cyst culture was collected and centrifuged at 2550× *g* for 10 min at 4 °C. The supernatant was removed, and the algal pellet was freeze dried. The pellet was then extracted with a mixture of cyclohexane/acetone/ethanol (CAE 2/1/1) for 48 h at room temperature. The solution was filtered, and the solvent phase was evaporated under vacuum. The carotenoid-enriched extract obtained was suspended in CAE (5 mL), and an aliquot (0.1 mL) was withdrawn and diluted in CAE (1 mL), then measured spectrophotometrically (UV/VIS/NIR, JASCO V-650, Tokyo, Japan) at 530 nm. NAst content in the carotenoid extract was determined using a calibration curve prepared with standard astaxanthin in the free form (0.5–25 μg mL^−1^). The entire extract was divided into sub-aliquots that were dried, kept in the dark to avoid degradation, and used for the biochemical assays.

### 4.3. Carotenoid-Enriched Extract Characterization

The aliquot of carotenoid-enriched extract in CAE was diluted in DMSO (0.08 mL) and methanol (0.4 mL) and analyzed by HPLC-UV-vis at 470 nm. Liquid chromatography analysis was performed using an HPLC system (Agilent 1200 series, Agilent Technologies Italia S.p.A, Cernusco sul Naviglio, Milan, Italy) coupled to a UV-vis diode array detector. The separation was performed using a XBridge C8 column, 137 Å, 3.5 μm, 4.6 mm × 150 mm (Waters, Milford, MA, USA), maintained at 30 °C, with an injected volume of 5 μL. The mobile phase was constituted as follows: H_2_O (solvent A) and methanol (solvent B).; chromatographic separation was achieved at a 0.7 mL min^−1^ flow rate under the following gradient elution conditions: from 80% of B (20% of A) to 100% of B (0% of A) from 0 to 10 min, 100% B from 10 to 18 min, from 100% of B (0% of A) to 80% of B (20% of A) from 18 to 20 min. All the changes in the mobile phase composition were linear. The qualitative identification of the astaxanthin monoesters in the extracts was carried out through HPLC-MS analyses performed on an Agilent 1260 Infinity II system coupled to an electrospray ionization mass spectrometer (positive-ion mode, m/z = 100–3000 amu, fragmentor 30 V). The column was the same as the one used for the HPLC/UV-Vis analysis; the mobile phase was modified by adding trifluoroacetic acid 0.1% *v/v* to both solvents. Chromatographic separation was achieved at a 0.4 mL min^−1^ flow rate under the following gradient elution conditions: 80–100% B from 0 to 10 min, 100% B from 10 to 30 min, 100–80% B from 30 to 32 min. Chemstation software was used for data processing.

### 4.4. Cell Culture

Neuroblastoma stem cells (SHSY-5Y) were cultured in Dulbecco Modified Eagle’s medium (DMEM) containing 4.5 g/L glucose, 2 mM glutamine, 1% penicillin/streptomycin, and 10% FBS (Fetal Bovine Serum). Cells were grown at 37 °C in 5% CO_2_ with saturating humidity. DMEM and FBS were purchased by Euroclone (Pero (MI), Italy).

All the other solvents and chemicals were purchased from Merck KGaA (Darmstadt, Germany) unless differently stated.

### 4.5. Cell Proliferation Assay

In order to exclude possible toxic effects of astaxanthin on cell growth, an MTT (3-(4,5-dimethylthiazol-2-yl)-2,5-diphenyltetrazolium bromide) assay was performed. A total of 3 × 10^3^ cells were seeded in a 96-well plate and cultured in control conditions to allow correct adhesion. After 24 h, the cells were treated with 3.7 ng/µL of astaxanthin, and proliferation was followed for 96 h. Every 24 h, growth medium was removed and replaced with 300 μM of MTT for 60 min. Then, formazan salts were solubilized in dimethyl sulfoxide (DMSO) for 15 min, and absorbance at 575 nm was detected using a plate reader (Enspire, Perkin Elmer).

Cell proliferation was assessed by MTT assay. SHSY-5Y cells were seeded in 96-well plates at 3 × 10^3^ cells/well in complete DMEM. Experiments were performed after 24 h of incubation at 37 °C in 5% CO2 to allow adhesion. After this time, cells were washed with HBSS and incubated with 3.7 ng/µL NAst in complete DMEM at different times. After washing them twice with HBSS buffer (156 mM NaCl, 3 mM KCl, 2 mM MgSO4, 1.25 mM KH2PO4, 2 mM CaCl2, 10 mM glucose, and 10 mM HEPES; pH adjusted to 7.4 with NaOH), the cells were incubated with 300 μM of MTT (((3-(4,5-dimethylthiazol-2-yl)-2,5-diphenyltetrazolium bromide)) in DMEM. The medium was removed after 60 minutes, and formazan salts were solubilized in dimethyl sulfoxide (DMSO) for 15 min. The absorbance of each well was measured at 575 nm using a plate reader (Enspire, Perkin Elmer, Waltham, MA, USA).

### 4.6. Measurement of Reactive Oxygen Species (ROS)

ROS intracellular levels were assessed using the reactive oxygen species probe 2,7-dichlorodihydrofluorescein diacetate (DCFH-DA, Thermo Fisher Scientific, Waltham, MA, USA), as described in Bergamini et al. [64]. Briefly, 15 × 10^3^ cells per well were seeded in 96-well plates (Optiplate, Perkin Elmer, Waltham, MA, USA) and cultured as described above. Then, the cells were incubated for 24 h with 3.7 ng/µL of astaxantin. Subsequently, the medium was removed and 10 μM of DCFH-DA in DMEM was added for 30 min. After washing them twice with HBSS, the cells were treated for 30 min with 130 μM of tert-butyl hydroperoxide (TBH). Finally, fluorescence (λexc = 485 nm; λem= 535 nm) was detected using a plate reader (Enspire, Perkin Elmer, Waltham, MA, USA). Data were normalized on protein contents obtained determined via Lowry assay.

### 4.7. Lipid Peroxidation Assay

Cell membrane peroxidation was assessed using the lipid peroxidation sensor BODIPY^®^ 581/591 (Thermo Fisher Scientific), following Pap et al. [65]. The oxidation of the polyunsaturated butadienyl portion of the dye results in a shift of the fluorescence emission peak from ~590 nm (red) to ~510 nm (green). Briefly, cells were seeded in a µ-Slide 8 Well (Ibidi, Germany), following the manufacturer’s instructions, and treated with 3.7 ng/µL astaxanthin for 24 h. After this time, the cells were stained with 1 μM of BODIPY^®^ 581/591 C11 in complete medium for 1 h. Then, the cells were carefully washed and treated for 30 min with 100 µM tert-butyl hydroperoxide or vehicle to induce oxidative stress. Images were acquired with a Nikon C1si confocal microscope (Nikon, Tokyo, Japan), and fluorescence intensity was analyzed using ImageJ software. At least 50 cells for each experimental condition were evaluated, and data were expressed as the ratios of green-to-red fluorescence intensities ± standard deviations.

### 4.8. Glutathione Analysis

Neuroblastoma stem cells were seeded in 96-well plates (OptiPlate Black; PerkinElmer, Waltham, MA, USA) at a density of 15 × 10^3^ cells/well in complete DMEM and incubated for 24 h to allow adhesion. After this time, the medium was removed, and cells were treated for 3 h with 3.7 ng/μL of NAst. Total glutathione was analyzed using the GSH/GSSG-GloTM kit (Promega), following the manufacturer’s instructions. Luminescence was detected using a multiplate reader (Enspire, Perkin Elmer, Waltham, MA, USA). Data were shown as the means ± standard errors of at least 3 independent experiments.

### 4.9. NQO1 Assay

Astaxanthin’s ability to induce the activity of NAD(P)H:quinone oxidoreductase 1 (DT-diaphorase, NQO1)—an enzyme that is able to accept electrons from NADH and reduce quinone—was tested. After treatment with astaxanthin, as described above, the cells were detached and lysed in Tris−HCl buffer containing bovine serum albumin (0.07%). The reaction was started by adding 200 μM NADH, and the absorbance increase at 340 nm was followed using a Jasco-V550 spectrophotometer (Jasco, Easton, MD, USequipped with a stirring device and a thermostatic control. Specific NQO1 activity was determined by inhibition with 20 μM of dicumarol. An extinction coefficient of 6.22 mM⁻^1^ cm⁻^1^ was used, and data were reported as fold increases in comparison with untreated cells ± SDs; *n* = 4, *p* ≤ 0.01.

### 4.10. Ferroptosis Assay

A ferroptosis assay was performed following Rizzardi et al., with minor modifications. A total of 10 × 10^3^ cells per well were seeded in a 96-well plate (Optiplate, Perkin Elmer Waltham, MA, USA) and treated as reported previously. Afterwards, different concentrations of erastin—an inhibitor of the system xc-cystine/glutamate antiporter—were added to the culture medium for 24 h. Then, the growth medium was removed, and cell viability was estimated by MTT assay. Absorbance at 575 nm was detected using a plate reader (Enspire, Perkin Elmer, Waltham, MA, USA).

### 4.11. qRT-PCR

SH-SYS5 cells were seeded and treated as described above. Then, cells were trypsinized and counted. A total of 5 × 10^6^ cells was centrifuged and washed twice with phosphate-buffered saline (PBS). Total RNA was isolated using a RNeasy Mini kit (Qiagen, Hilden, Germany), according to the manufacturer’s protocol. RNA was quantified using a NanoDrop spectrophotometer (Thermo Fisher Scientific, Waltham, MA, USA), and integrity was checked on a 2% agarose gel. A quantity of 1 µg of RNA was employed to generate cDNA using the Wonder RT – cDNA- Synthesis Kit (EuroClone, Milano, Italy). cDNA was used to analyze the levels of transcripts by quantitative real-time PCR (qRT-PCR). The Tli RNase H Plus (Diateh labline, Ancona, Italy) and the LightCycler 2.0 instrument (Roche, Basel, Switzerland) were employed. The first gene analyzed was the house-keeping gene actin, and its CT values were used as standards in the ΔΔCT method of results analysis. cDNA samples from SH-SYS5-treated cells were compared to control cells. Samples were analyzed at least in triplicate and the Student’s *t*-test was used to verify the significance of results (a *p*-value less than 0.05 was considered significant). Primers were purchased from Sigma-Aldrich, and their sequences and conditions are listed in Table 1.

### 4.12. Quantitative Phase Imaging (QPI) Microscopy

A quantitative phase image (QPI) microscopy assay was performed using the Livecyte microscope (Phase Focus Limited, Sheffield, UK), according to the manufacturer’s indications. In brief, SHSY-5Y cells were seeded in a 96-well plate (Sarsted, Milan, Italy) at 10 × 10^3^/well. After 24 h of incubation, the cells were treated with 4 ng/µL of NAst or 4 ng/µL of synthetic astaxanthin in complete DMEM. After 24 h, RSL3, a ferroptosis-inducing agent, was added to the culture medium at a concentration of 4 µM. Images were acquired every 60 min for 3 days using a 10× objective (0.25 NA) at 37 °C and 5% CO_2_. Data were analyzed using the Cell Analysis Toolbox software (Phase Focus Limited, Sheffield, UK) to evaluate cell growth, cell motility, and morphology.

### 4.13. Statistical Analysis

Statistical significance was evaluated using the unpaired Student’s *t*-test (single comparisons) or one-way ANOVA followed by Bonferroni (multiple comparisons) testing. The analysis was carried out using the GraphPad Prism software package (GraphPad Software, San Diego, CA, USA). The number n refers to the number of biological replicates. *p* values ≤ 0.05 were considered statistically significant. Statistical analysis was performed using GraphPad software.

## Figures and Tables

**Table 1:** Primer sequences.

PRIMER	SEQUENCE	T_annealing_	T_fluorescence_
***βactin* FW**	CCAACCGCGAGAAGATGA	55 °C	80 °C
***βactin* RV**	CCAGAGGCGTACAGGGATAG		
***NRF2* FW**	CATTCCCGAATTACAGTGTC	60 °C	75 °C
***NRF2* RV**	GGAGATCGATGAGTAAAAATGG		
***GSR* FW**	GACCTATTCAACGAGCTTTAC	60 °C	75 °C
***GSR* RV**	CAACCACCTTTTCTTCCTTG		
***TXNRD1 FW***	AGACAGTTAAGCATGATTGG	60 °C	75 °C
***TXNRD1 RV***	AATTGCCCATAAGCATTCTC		
***CAT* FW**	CAACAAAGTGCAAGATTCTG	55 °C	80 °C
***CAT* REV**	TGCATTCACATGGCATAAAG		

## Data Availability

Not applicable.

## References

[1] Seabra L.M.J., Pedrosa L.F.C. (2010). Astaxanthin: Structural and functional aspects. Rev. Nutr..

[2] Begum H., Yusoff F.M.D., Banerjee S., Khatoon H., Shariff M. (2016). Availability and Utilization of Pigments from Microalgae. Crit. Rev. Food Sci. Nutr..

[3] Miki W. (1991). Biological functions and activities of animal carotenoids. Pure Appl. Chem..

[4] Palozza P., Krinsky N.I. (1992). Astaxanthin and canthaxanthin are potent antioxidants in a membrane model. Arch. Biochem. Biophys..

[5] Hussein G., Sankawa U., Goto H., Matsumoto K., Watanabe H. (2006). Astaxanthin, a carotenoid with potential in human health and nutrition. J. Nat. Prod..

[6] Ambati R.R., Moi P.S., Ravi S., Aswathanarayana R.G. (2014). Astaxanthin: Sources, extraction, stability, biological activities and its commercial applications—A review. Mar. Drugs.

[7] Paniagua-Michel J. (2015). Microalgal Nutraceuticals. Handbook of Marine Microalgae: Biotechnology Advances.

[8] Lakey-Beitia J., Jagadeesh Kumar D., Hegde M.L., Rao K.S. (2019). Carotenoids as novel therapeutic molecules against neurodegenerative disorders: Chemistry and molecular docking analysis. Int. J. Mol. Sci..

[9] Régnier P., Bastias J., Rodriguez-Ruiz V., Caballero-Casero N., Caballo C., Sicilia D., Fuentes A., Maire M., Crepin M., Letourneur D. (2015). Astaxanthin from Haematococcus pluvialis prevents oxidative stress on human endothelial cells without toxicity. Mar. Drugs.

[10] Galasso C., Orefice I., Pellone P., Cirino P., Miele R., Ianora A., Brunet C., Sansone C. (2018). On the neuroprotective role of astaxanthin: New perspectives?. Mar. Drugs.

[11] Fassett R.G., Coombes J.S. (2011). Astaxanthin: A potential therapeutic agent in cardiovascular disease. Mar. Drugs.

[12] Davinelli S., Nielsen M.E., Scapagnini G. (2018). Astaxanthin in skin health, repair, and disease: A comprehensive review. Nutrients.

[13] Zuluaga M., Gueguen V., Letourneur D., Pavon-Djavid G. (2018). Astaxanthin-antioxidant impact on excessive Reactive Oxygen Species generation induced by ischemia and reperfusion injury. Chem. Biol. Interact..

[14] Liu X., Shibata T., Hisaka S., Osawa T. (2009). Astaxanthin inhibits reactive oxygen species-mediated cellular toxicity in dopaminergic SH-SY5Y cells via mitochondria-targeted protective mechanism. Brain Res..

[15] Capelli B., Bagchi D., Cysewski G.R. (2013). Synthetic astaxanthin is significantly inferior to algal-based astaxanthin as an antioxidant and may not be suitable as a human nutraceutical supplement. Nutrafoods.

[16] Panis G., Carreon J.R. (2016). Commercial astaxanthin production derived by green alga *Haematococcus pluvialis*: A microalgae process model and a techno-economic assessment all through production line. Algal Res..

[17] Nguyen K. (2013). Astaxanthin: A comparative case of synthetic vs. natural production. Chem. Biomol. Eng. Publ. Other Works.

[18] Higuera-Ciapara I., Félix-Valenzuela L., Goycoolea F.M. (2006). Astaxanthin: A review of its chemistry and applications. Crit. Rev. Food Sci. Nutr..

[19] Shah M.R., Liang Y., Cheng J.J., Daroch M. (2016). Astaxanthin-Producing Green From Single Cell to High Value Commercial Products. Front. Plant Sci..

[20] Todd Lorenz R. (2000). Astaxanthin, nature’s super carotenoid. Cynotech Corp..

[21] Johnson E.A., An G.H. (1991). Astaxanthin from microbial sources. Crit. Rev. Biotechnol..

[22] Roy S.S., Pal R. (2015). Microalgae in Aquaculture: A Review with Special References to Nutritional Value and Fish Dietetics. Proc. Zool. Soc..

[23] Industry Experts (2015). Global Astaxanthin Market: Sources, Technologies and Applications.

[24] Brendler T., Williamson E.M. (2019). Astaxanthin: How much is too much? A safety review. Phyther. Res..

[25] Stachowiak B., Szulc P. (2021). Astaxanthin for the food industry. Molecules.

[26] Turck D., Castenmiller J., de Henauw S., Hirsch-Ernst K.I., Kearney J., Maciuk A., Mangelsdorf I., McArdle H.J., Naska A., Pelaez C. (2020). Safety of astaxanthin for its use as a novel food in food supplements. EFSA J..

[27] Misawa N., Satomi Y., Kondo K., Yokoyama A., Kajiwara S., Saito T., Ohtani T., Miki W. (1995). Structure and functional analysis of a marine bacterial carotenoid biosynthesis gene cluster and astaxanthin biosynthetic pathway proposed at the gene level. J. Bacteriol..

[28] Yokoyama A., Miki W. (1995). Composition and presumed biosynthetic pathway of carotenoids in the astaxanthin-producing bacterium *Agrobacterium aurantiacum*. FEMS Microbiol. Lett..

[29] Liu J., Sun Z., Gerken H., Liu Z., Jiang Y., Chen F. (2014). Chlorella zofingiensis as an alternative microalgal producer of astaxanthin: Biology and industrial potential. Mar. Drugs.

[30] Boussiba S., Vonshak A. (1991). Astaxanthin accumulation in the green alga haematococcus pluvialis. Plant Cell Physiol..

[31] Boussiba S. (2000). Carotenogenesis in the green alga Haematococcus pluvialis: Cellular physiology and stress response. Physiol. Plant..

[32] Liu B.J., van der Meer J.P., Zhang L., Zhang Y. (2017). Cultivation of haematococcus pluvialis for astaxanthin production. Microalgal Production for Biomass and High-Value Products.

[33] Tripathi U., Sarada R., Ramachandra Rao S., Ravishankar G.A. (1999). Production of astaxanthin in Haematacoccus pluvialis cultured in various media. Bioresour. Technol..

[34] Li J., Zhu D., Niu J., Shen S., Wang G. (2011). An economic assessment of astaxanthin production by large scale cultivation of Haematococcus pluvialis. Biotechnol. Adv..

[35] Lorenz R.T., Cysewski G.R. (2000). Commercial potential for *Haematococcus microalgae* as a natural source of astaxanthin. Trends Biotechnol..

[36] Park J.S., Chyun J.H., Kim Y.K., Line L.L., Chew B.P. (2010). Astaxanthin decreased oxidative stress and inflammation and enhanced immune response in humans. Nutr. Metab..

[37] Gite S., Ross R.P., Kirke D., Guihéneuf F., Aussant J., Stengel D.B., Dinan T.G., Cryan J.F., Stanton C. (2019). Nutraceuticals to promote neuronal plasticity in response to corticosterone-induced stress in human neuroblastoma cells. Nutr. Neurosci..

[38] Liu X., Osawa T. (2009). Astaxanthin protects neuronal cells against oxidative damage and is a potent candidate for brain food. Forum Nutr..

[39] Yan T., Zhao Y., Zhang X., Lin X. (2016). Astaxanthin inhibits acetaldehyde-induced cytotoxicity in SH-SY5Y cells by modulating Akt/CREB and p38MAPK/ERK signaling pathways. Mar. Drugs.

[40] Liu X., Osawa T. (2007). Cis astaxanthin and especially 9-cis astaxanthin exhibits a higher antioxidant activity in vitro compared to the all-trans isomer. Biochem. Biophys. Res. Commun..

[41] Ikeda Y., Tsuji S., Satoh A., Ishikura M., Shirasawa T., Shimizu T. (2008). Protective effects of astaxanthin on 6-hydroxydopamine-induced apoptosis in human neuroblastoma SH-SY5Y cells. J. Neurochem..

[42] Li Z., Dong X., Liu H., Chen X., Shi H., Fan Y., Hou D., Zhang X. (2013). Astaxanthin protects ARPE-19 cells from oxidative stress via upregulation of Nrf2-regulated phase II enzymes through activation of PI3K/AkT. Mol. Vis..

[43] Li J., Cao F., Yin H., Huang Z.J., Lin Z.T., Mao N., Sun B., Wang G. (2020). Ferroptosis: Past, present and future. Cell Death Dis..

[44] Yang W.S., Stockwell B.R. (2016). Ferroptosis: Death by Lipid Peroxidation. Trends Cell Biol..

[45] Sui X., Zhang R., Liu S., Duan T., Zhai L., Zhang M., Han X., Xiang Y., Huang X., Lin H. (2018). RSL3 drives ferroptosis through GPX4 inactivation and ros production in colorectal cancer. Front. Pharmacol..

[46] Sun Y., Deng R., Zhang C. (2020). Erastin induces apoptotic and ferroptotic cell death by inducing ROS accumulation by causing mitochondrial dysfunction in gastric cancer cell HGC.27. Mol. Med. Rep..

[47] Deroche M.E., Briantais J.M. (1974). Absorption Spectra of Chlorophyll forms, β-Carotene and Lutein in Freeze-Dried chloroplasts. Photochem. Photobiol..

[48] Drummen G.P.C., Van Liebergen L.C.M., Op den Kamp J.A.F., Post J.A. (2002). C11-BODIPY581/591, an oxidation-sensitive fluorescent lipid peroxidation probe: (Micro)spectroscopic characterization and validation of methodology. Free Radic. Biol. Med..

[49] Stockwell B.R., Friedmann Angeli J.P., Bayir H., Bush A.I., Conrad M., Dixon S.J., Fulda S., Gascón S., Hatzios S.K., Kagan V.E. (2017). Ferroptosis: A Regulated Cell Death Nexus Linking Metabolism, Redox Biology, and Disease. Cell.

[50] Tang D., Chen X., Kang R., Kroemer G. (2021). Ferroptosis: Molecular mechanisms and health implications. Cell Res..

[51] Hayes J.D., Dinkova-Kostova A.T. (2014). The Nrf2 regulatory network provides an interface between redox and intermediary metabolism. Trends Biochem. Sci..

[52] Fakhri S., Abbaszadeh F., Dargahi L., Jorjani M. (2018). Astaxanthin: A mechanistic review on its biological activities and health benefits. Pharmacol. Res..

[53] Kumar S., Kumar R., Kumari A., Panwar A. (2021). Astaxanthin: A super antioxidant from microalgae and its therapeutic potential. J. Basic Microbiol..

[54] Zhang J., Ding C., Zhang S., Xu Y. (2020). Neuroprotective effects of astaxanthin against oxygen and glucose deprivation damage via the PI3K/Akt/GSK3β/Nrf2 signalling pathway in vitro. J. Cell. Mol. Med..

[55] Wang H.Q., Sun X.B., Xu Y.X., Zhao H., Zhu Q.Y., Zhu C.Q. (2010). Astaxanthin upregulates heme oxygenase-1 expression through ERK1/2 pathway and its protective effect against beta-amyloid-induced cytotoxicity in SH-SY5Y cells. Brain Res..

[56] Shin J., Kim J.E., Pak K.J., Kang J.I., Kim T.S., Lee S.Y., Yeo I.H., Park J.H.Y., Kim J.H., Kang N.J. (2017). A combination of soybean and Haematococcus extract alleviates ultraviolet B-induced photoaging. Int. J. Mol. Sci..

[57] Sayo T., Sugiyama Y., Inoue S. (2013). Lutein, a nonprovitamin A, activates the retinoic acid receptor to induce HAS3-dependent hyaluronan synthesis in keratinocytes. Biosci. Biotechnol. Biochem..

[58] Ni Y., Nagashimada M., Zhuge F., Zhan L., Nagata N., Tsutsui A., Nakanuma Y., Kaneko S., Ota T. (2015). Astaxanthin prevents and reverses diet-induced insulin resistance and steatohepatitis in mice: A comparison with Vitamin E. Sci. Rep..

[59] Jiang X., Stockwell B.R., Conrad M. (2021). Ferroptosis: Mechanisms, biology and role in disease. Nat. Rev. Mol. Cell Biol..

[60] Ren J.X., Sun X., Yan X.L., Guo Z.N., Yang Y. (2020). Ferroptosis in Neurological Diseases. Front. Cell. Neurosci..

[61] Brotosudarmo T.H.P., Limantara L., Setiyono E. (2020). Heriyanto Structures of Astaxanthin and Their Consequences for Therapeutic Application. Int. J. Food Sci..

[62] Villaró S., Ciardi M., Morillas-españa A., Sánchez-zurano A., Acién-fernández G., Lafarga T. (2021). Microalgae derived astaxanthin: Research and consumer trends and industrial use as food. Foods.

[63] Pezzolesi L., Peña V., Le Gall L., Gabrielson P.W., Kaleb S., Hughey J.R., Rodondi G., Hernandez-Kantun J.J., Falace A., Basso D. (2019). Mediterranean *Lithophyllum stictiforme* (*Corallinales*, *Rhodophyta*) is a genetically diverse species complex: Implications for species circumscription, biogeography and conservation of coralligenous habitats. J. Phycol..

[64] Bergamini C., Moruzzi N., Sblendido A., Lenaz G., Fato R. (2012). A water soluble CoQ 10 formulation improves intracellular distribution and promotes mitochondrial respiration in cultured cells. PLoS ONE.

[65] Pap E.H.W., Drummen G.P.C., Winter V.J., Kooij T.W.A., Rijken P., Wirtz K.W.A., Op Den Kamp J.A.F., Hage W.J., Post J.A. (1999). Ratio-fluorescence microscopy of lipid oxidation in living cells using C11-BODIPY(58l/591). FEBS Lett..

